# Design of an Electrochemical Cell for Continuous Wave EPR Measurements of Radical Ions

**DOI:** 10.1002/chem.202402719

**Published:** 2024-10-29

**Authors:** Dominic K. F. Bruyers, Sabine Richert

**Affiliations:** ^1^ Institute of Physical Chemistry Albertstraße 21 79104 Freiburg Germany

**Keywords:** radical ions, EPR spectroscopy, molecular electrochemistry, spectroelectrochemistry, reactive intermediates

## Abstract

The combination of continuous wave electron paramagnetic resonance (cw‐EPR) with electrochemistry is highly attractive as it allows a clean in‐situ generation and the subsequent spectroscopic characterisation of radical ions, which are important intermediates in many photocatalytic cycles as well as light‐induced processes occurring in biological systems or optoelectronic devices. Although commercial setups for spectroelectrochemical EPR are available, they are often expensive and tailored to a particular spectroscopic setup. Here we present a design for a low‐cost electrochemical EPR cell that can be used in combination with any commercial cw‐EPR instrumentation. The cell design is compared to existing setups and the performance of the cell is evaluated by comparison of EPR spectra obtained by chemical and electrochemical oxidation of a graphene fragment.

## Graphical abstract

The combination of continuous wave EPR and electrochemistry is powerful for the characterisation of radical ions, which are important intermediates in many processes of biological or technological relevance. Here, we present a simple design for an electrochemical EPR cell that can be replicated easily, at low cost, and is compatible with any standard cwEPR instrumentation including benchtop spectrometers.

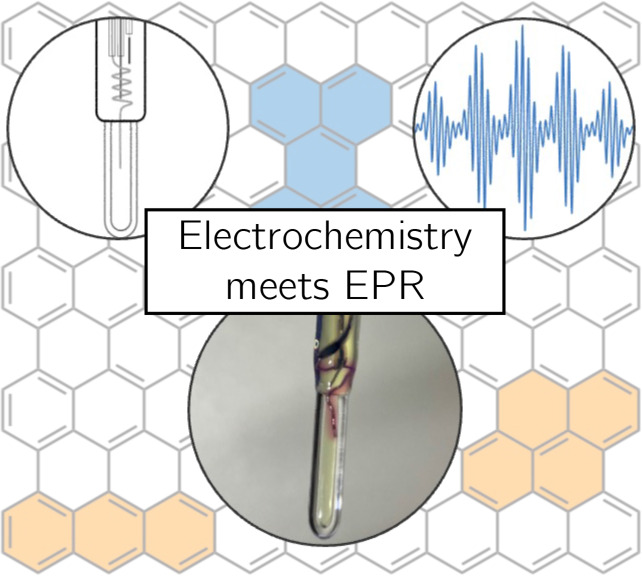

## Introduction

Combining electron paramagnetic resonance (EPR) spectroscopy and electrochemistry, the properties of paramagnetic species generated by oxidation or reduction can be investigated.[^1^, ^2^, ^3^, ^4^] Such paramagnetic species are intermediates, for instance, in catalytic cycles[^5^, ^6^] or biological processes (e. g. photosynthesis),[^7^, ^8^] but the combination of methods is also of interest for various applications in the field of materials science (e. g. batteries, optoelectronic devices).[^9^, ^10^] With EPR spectroscopy, paramagnetic species can be detected (quantitatively) and characterised. The technique can be used to obtain information on the electronic structure in the proximity of the unpaired electron spin, the delocalisation of the spin density, and help to clarify different oxidation states and the coordination geometry of transition metals.[^5^, ^10^]

Radical ions can be generated either by electrochemical or chemical oxidation or reduction. The generation of radical ions by chemical methods is typically more accessible, as it does not require a sophisticated experimental setup, but has some disadvantages compared to the electrochemical way: The added redox agents are frequently paramagnetic themselves. Their EPR spectra may overlap with the species to be analysed, complicating the analysis. The counter ions, present in the mixture, may also alter the localisation of the spin density in an unwanted way. In cases where multiple oxidation or reduction steps are possible, it can be difficult to control the reaction progress using chemical methods, resulting in a undefined mixture of different species.^[11]^

Electrochemical approaches are attractive, since sample preparation is easier and faster. There is no need to add redox agents, which enables an in‐situ monitoring of reactions. By applying a well‐defined potential, the turnover of oxidation or reduction can be controlled precisely and multiple oxidation/reduction steps can potentially be avoided.[^12^, ^13^] However, spectroelectrochemical EPR (SEC‐EPR) requires a special experimental setup, which may be expensive, difficult to handle, or require additional expertise, making the method generally less accessible.

As different samples require different measurement conditions, a variety of experimental setups for SEC‐EPR have already been reported in the literature.[^10^, ^14^] Most experimental approaches are generally similar, but optimised for specific sample conditions (e. g. solution vs. film),[^6^, ^7^] different types of measurements (e. g. continuous wave vs. pulse EPR),[^5^, ^9^, ^10^] or different EPR equipment (e. g. X‐band vs. Q/W‐band EPR).^[15]^

The design of a suitable SEC‐EPR cell is challenging, as the ideal measurement conditions for EPR and electrochemical measurements are fundamentally different. Room temperature cw‐EPR measurements are best performed in solvents with low dielectric constants to limit the absorption of microwaves (/maximise the sensitivity). However, electrochemical measurements often require the use of electrolyte solutions with high permittivity. To unite these requirements, it is crucial to reduce the sample volume and to place the sample in the electric field minimum of the resonator.^[10]^ Frequently, this is realised by using flat cells or capillaries. Further, the design of the electrodes is critical, as the introduction of metal into the resonator can compromise the sensitivity of the EPR experiment substantially. To limit any additional reflection or absorption of microwaves, two strategies are commonly employed: either the electrodes are placed outside the active zone of the resonator, which usually requires a flow system,[^5^, ^10^, ^14^] or, when placing the electrodes inside the resonator cavity, very thin wires or meshes are employed instead of bulky electrodes.[^10^, ^13^, ^15^, ^16^]

Here, we describe the design of a SEC‐EPR cell for continuous wave (cw) EPR experiments at X‐band frequencies. This home‐built cell can be built at low cost with commercially available materials. The cell design was kept as simple as possible so that the cell is robust, easy to handle, and easy to replicate with access to a glassblower. The cell design presented here has only a small effect on the quality (Q) factor of the EPR cavity and therefore little impact on the sensitivity of the EPR measurement. This allows the acquisition of high‐quality EPR spectra even on bench‐top EPR instruments. To illustrate the performance of the SEC‐EPR cell, we compare EPR spectra of a triangulene‐derivative obtained after chemical and electrochemical oxidation.

## Cell Design and Characterisation

**Figure 1:**
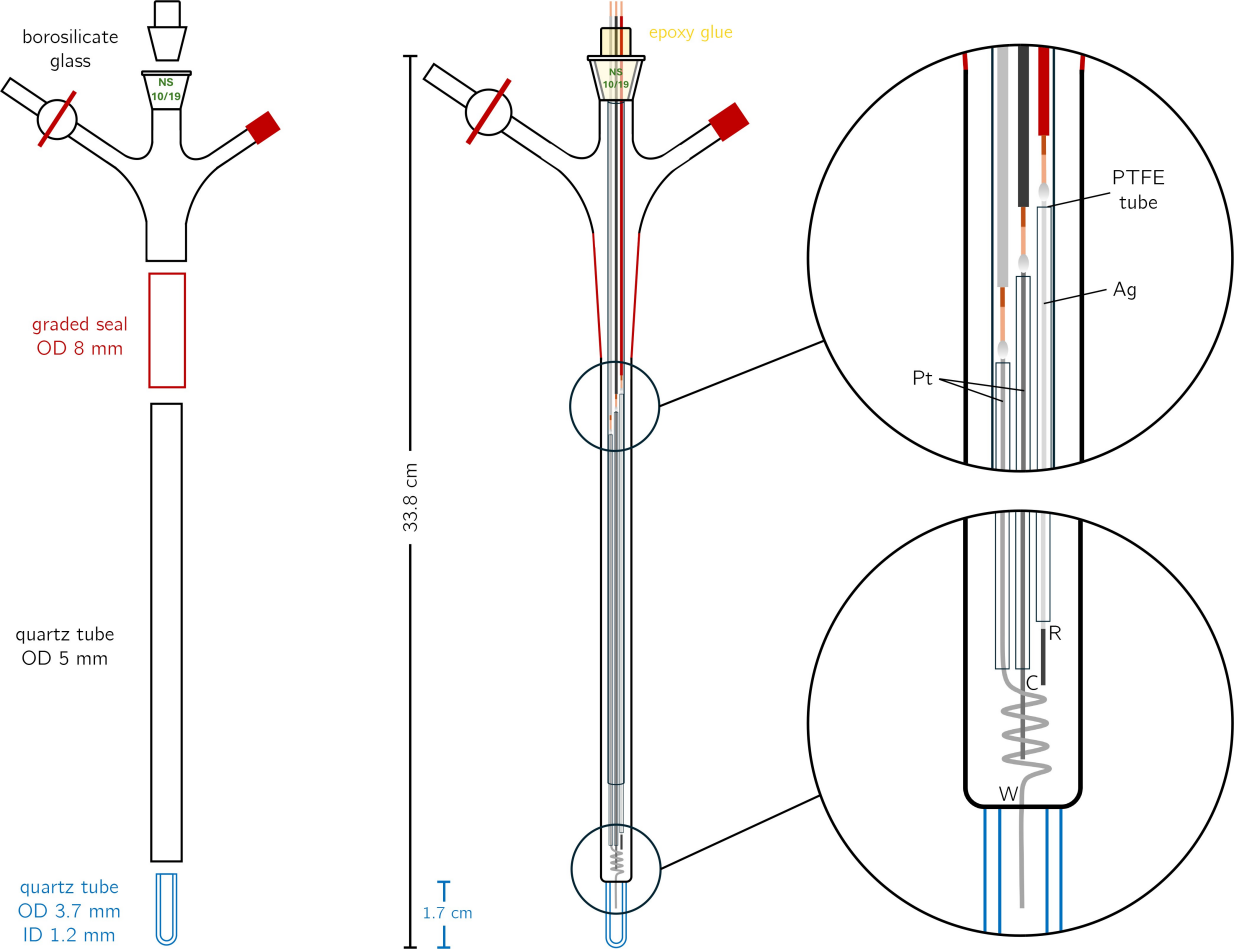
Illustration of the cell design. Left: individual, commercially‐available, parts of the cell. Centre: cw‐EPR cell with electrodes inserted. Right: zoom on individual parts.

Figure 1 shows a schematic representation of the SEC‐EPR cell. The upper part of the cell is made of borosilicate glass and is commercially available. There are three inlets: the rightmost inlet is used for the connection to a Schlenk line. The central inlet is a ground glass outer joint, size 10 (NS 10/19), and serves to insert the electrodes. The third connection is not required for the work described here. To connect this upper part of the SEC‐EPR cell, made of laboratory glass, to an EPR tube made of quartz glass, a graded seal with an outer diameter of ~8 mm was used.

The quartz tube, that forms the lower part of the cell, had a length of about 20 cm, an outer diameter of 5 mm, and an inner diameter of 4 mm. The relatively long tube is required for the operation of the cell in combination with the standard resonator of a Bruker EMXnano benchtop EPR spectrometer. The tube diameters were chosen as they are compatible with standard EPR equipment and offer ample space to accommodate the electrodes. The very bottom part of the cell consists of a short quartz tube with a length of ~1.7 cm, an outer diameter of 3.7 mm, and an inner diameter of 1.2 mm. By using such a thick‐walled quartz tube at the bottom of the cell, the sample volume is minimised without affecting the filling factor of the cavity significantly.

The hook‐up wires (insulated copper wires, 1 mm diameter) for the connection of the electrodes, were fixed into a hollow ground glass inner joint to be inserted into the central inlet of the cell for the measurements. For this purpose, the three wires where held in place while the hollow joint was filled with a two‐component glass glue (epoxy), providing an air‐tight seal. The hook‐up wires reaching into the cell were then cut at different lengths between 10 and 20 cm. Subsequently, the insulating material at the bottom part of the hook‐up wires was removed and the copper wires were cleaned before soldering them to the Pt‐ and Ag‐wires forming the electrodes. Platium wires, with a a diameter of 0.5 mm, were used as the working and counter electrodes, while a silver wire with a diameter of 0.8 mm was used as pseudo‐reference electrode.

To avoid any contact between the electrodes inside the cell, they were insulated by inserting them into matching PTFE tubing, normally used for high performance liquid chromatography. To keep all wires inside the tube straight and in place over a length of more than 20 cm, they were guided by a thin‐walled 3 mm quartz tube, ending shortly before the bottom part of the cell (above the sample solution). Below this point, the protective teflon tubing was removed from the electrodes. In order to increase the surface area of the working electrode, compared to the counter electrode, the respective Pt‐wire was slightly coiled to increase the surface of the working electrode, with the end reaching into the constricted part of the sample tube as shown in Figure 1. The pseudo‐reference electrode was prepared according to Ref. [17].

The SEC‐EPR cell is inserted into the EPR resonator such that only the bottom part, with the reduced inner tube diameter (blue part in Figure 1), reaches into the active part of the resonator. The hook‐up wires at the top of the cell may then be connected to a potentiostat using crocodile clips.

To determine how the sensitivity of the EPR measurement is influenced by the cell, the quality‐factor Q of the EPR cavity was determined for different scenarios. Table 1 provides an overview of the Q‐factors obtained on a Bruker EMXnano benchtop spectrometer. The indicated values are average values of 16 measurements performed at different orientations of the cell and the electrodes. It can be seen that the cell itself, including the electrodes, only has a very minor influence on the Q‐factor, which is reduced by less than 3 %. The measurement sensitivity will thus essentially depend on the choice of the electrolyte solution, but very reasonable Q‐factors are obtained even when choosing polar solvents, such as THF, as the sample volume inside the resonator is comparatively low.

**Table 1:** Overview of the influence of the electrodes and the electrolyte solution on the Q‐factor of the resonator cavity measured on a Bruker EMXnano benchtop spectrometer. A 100 mM solution of tetrabutylammonium hexafluorophospate in tetrahydrofuran was chosen as the electrolyte.

	empty	electrodes	electrodes + electrolyte
Q_av_	6031	5890	4528

## Experimental Details

Before the measurements, stock solutions of the analytes and the electrolyte were prepared. A 200 mM solution of tetrabutylammonium hexafluorophospate (TBAHFP) in dry tetrahydrofuran (THF) was used as the electrolyte solution. The solvent, THF, was dried over molecular sieves (4 Å). The supporting electrolyte, TBAHFP, was dried by melting the solid under vacuum and subsequently stored under inert gas (Ar).

The analyte stock solutions were prepared at a concentration of 0.4 mM to 0.5 mM (0.25 mM for perylene due to solubility issues) in dry THF. To eliminate oxygen, the solutions were degassed by bubbling with argon gas for at least 5 minutes.

### EPR Spectroscopy

The samples for the EPR measurements were prepared by mixing the stock solutions of the analyte and the electrolyte in a 1 : 1 ratio under inert gas atmosphere, using roughly 0.2 mL of each solution. The resulting concentrations are 0.2 to 0.25 mM for the analytes (0.125 mM for perylene) and 100 mM for the supporting electrolyte TBAHFP.

The EPR cell was flushed with THF‐saturated argon gas for at least five minutes before the sample solution was added under inert gas conditions. In addition, solvent‐saturated argon gas was bubbled through the sample solution inside the cell for one minute. The cell was then sealed by insertion of the ground glass joint containing the electrodes.

The EPR measurements were carried out at room temperature on a Bruker EMXnano benchtop spectrometer. As mentioned above, the cell was inserted into the EPR resonator such that only the bottom part, with the reduced inner diameter, reached into the active part of the resonator, and subsequently connected to a PalmSens EmStat3+ potentiostat.

Before the start of the EPR experiment, the initial potential, measured directly when connecting the electrodes, was verified. If this initial potential was not within the expected range (0–200 mV in our case), the experiment was repeated after cleaning the electrodes. The potential was then set to a value close to the initial potential and held constant for three minutes before a cw‐EPR spectrum was recorded. If no radical signal was observed, the potential was increased by 100 mV and held again constant for three minutes before another scan was recorded. The procedure was repeated until the onset of a signal was observed.

The EPR spectra were recorded using a microwave power of 1 mW (20 dB attenuation) and a modulation amplitude of 0.02 mT. The recorded, background‐corrected, spectra were frequency‐corrected to 9.75 GHz and field‐corrected using a carbon fibre standard with g=2.002644 .^[18]^

After each measurement, the EPR‐cell and the electrodes were cleaned carefully to avoid contamination. The cell was cleaned by rinsing it alternately with water and ethanol and then dried by flushing it with inert gas from the bottom to the top. The reference electrode was cleaned by sanding down the top layer of the silver wire with commercially available sandpaper. Subsequently, the wire was immersed in a 0.1 M solution of sodium hypochlorite for about 15 minutes to refresh the silver chloride layer. The platinum counter and working electrodes were cleaned by burning off any residues that could not be washed off when rinsing the electrodes with ethanol and water.

## Results and Discussion

To verify the performance of the cell, EPR spectra were recorded for radical ions of several different literature‐known molecules, focussing on the anions of aromatic hydrocarbons such as perylene, pyrene, and anthracene.

### Comparison to Literature Data

**Figure 2:**
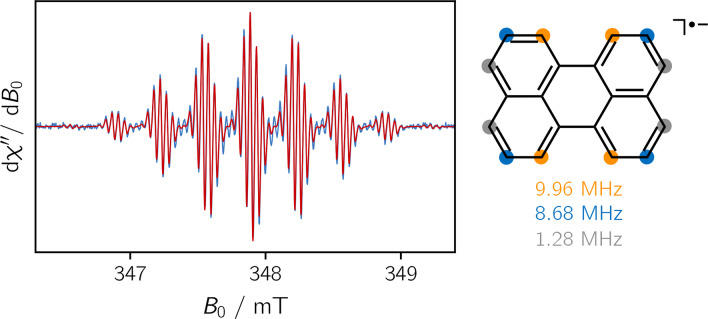
Experimental cw‐EPR spectrum of perylene (*left*) together with the corresponding numerical simulation. By comparison with DFT calculations, the measured isotropic hyperfine coupling constants can be assigned to the individual proton nuclei of the molecular structure (*right*). The proton hyperfine couplings obtained from the fit of the experimental data are indicated underneath the structure.

The perylene chromophore has three sets of four chemically inequivalent protons, which should result in a total of 125 lines in a cw‐EPR spectrum. However, many of these lines are not resolved due to spectral overlap. The EPR spectrum of the perylene anion is shown in Figure 2 and is in very good agreement with previous literature results.^[19]^ A numerical simulation of the measured spectrum using the EasySpin software package^[20]^ yields an isotropic *g*‐value of g=2.0027 and hyperfine couplings of 9.96 MHz (4×H), 8.68 MHz (4×H), and 1.28 MHz (4×H), for the three sets of protons. An assignment of these couplings to the individual ^1^H‐nuclei of the structure is also shown in Figure 2.

**Figure 3:**
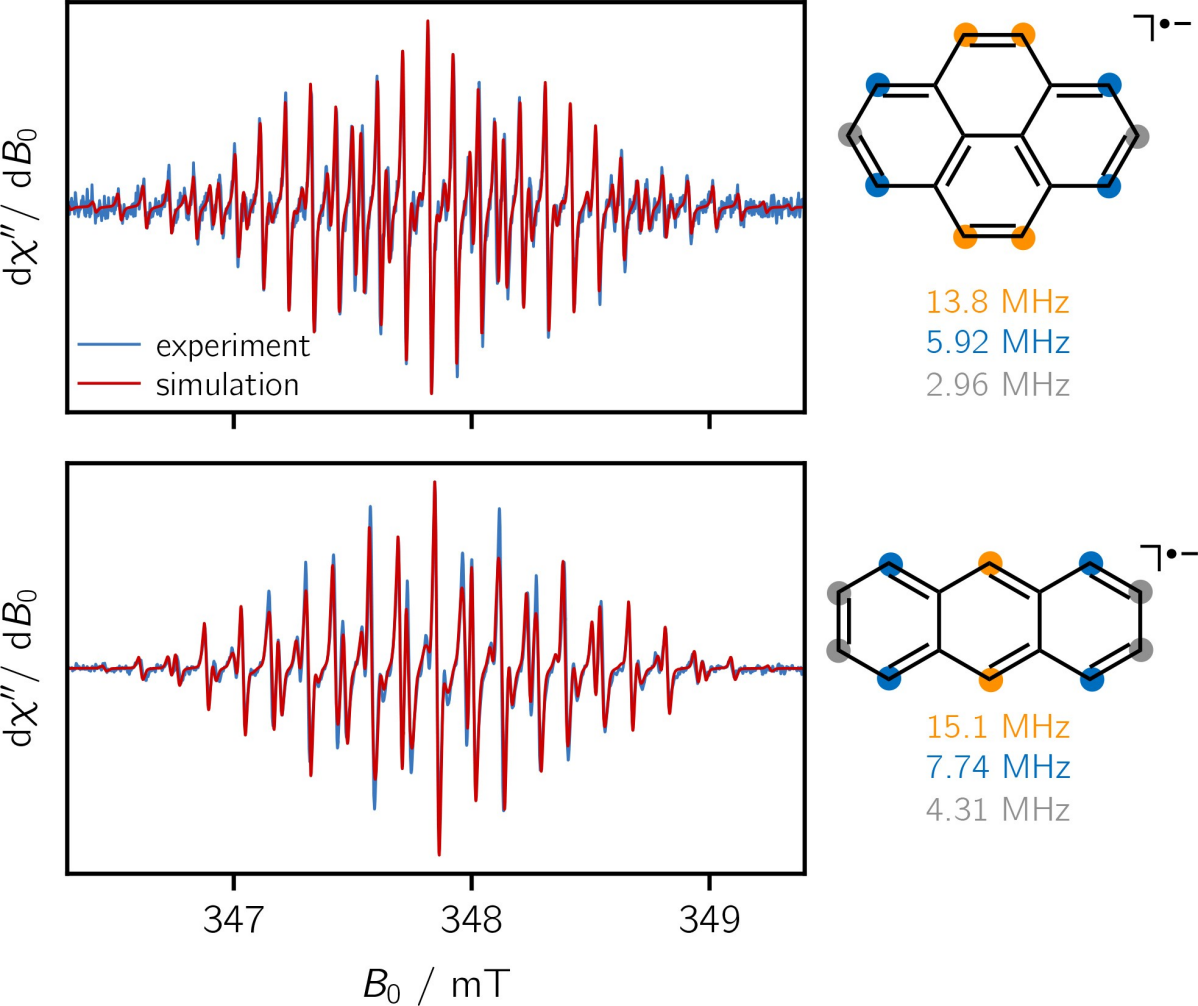
Measured cw‐EPR spectra of pyrene (*top*) and anthracene (*bottom*) together with numerical simulations of the spectra and an assignment of the experimentally determined couplings to the protons of the respective structures.

Further EPR experiments were performed with anthracene and pyrene as shown in Figure 3. The hyperfine coupling constants of the anions, obtained from a numerical simulation of the spectra, are again in very good agreement with known literature values.^[19]^ For pyrene, an isotropic *g*‐value of g=2.0028 and hyperfine couplings of 13.8 MHz (4×H), 5.92 MHz (4×H), and 2.96 MHz (2×H) were obtained, while the simulation for the anthracene anion yielded g=2.0024 and proton couplings of 15.1 MHz (2×H), 7.74 MHz (4×H), and 4.31 MHz (4×H). The assignment of these couplings to the individual proton nuclei is shown graphically in Figure 3.

The results demonstrate that the literature data can be reproduced well using a relatively simple cell design and standard experimental conditions. In addition, the measurements can be performed with little effort in a short amount of time. The EPR data above were acquired in only a few minutes, indicating that the material turnover is sufficient and that high‐quality data can be obtained.

### Comparison between Chemical and Electrochemical Oxidation

To illustrate the use of the SEC‐EPR cell for the investigation of novel materials of significant current interest, we reproduced the cw‐EPR spectrum of a triangulene‐derivative published recently by Valenta et al.,^[11]^ making use of electrochemical oxidation instead of chemical oxidation. Triangulene, also referred to as Clar's hydrocarbon, is the smallest non‐Kekulé graphene fragment composed of six benzenoid rings fused in a triangular shape. Persistent triangulene was a long sought‐after goal since the pioneering work of Erich Clar almost 70 years ago,^[21]^ possesses two unpaired electrons forming a triplet ground state, and may find applications in the field of organic spintronics.[^22^, ^23^]

In the investigated triangulene derivative, kinetic stablisation is achieved via the installment of three sterically demanding mesityl substituents. Full details on the synthesis and characterisation can be found in Ref. [11].

**Figure 4:**
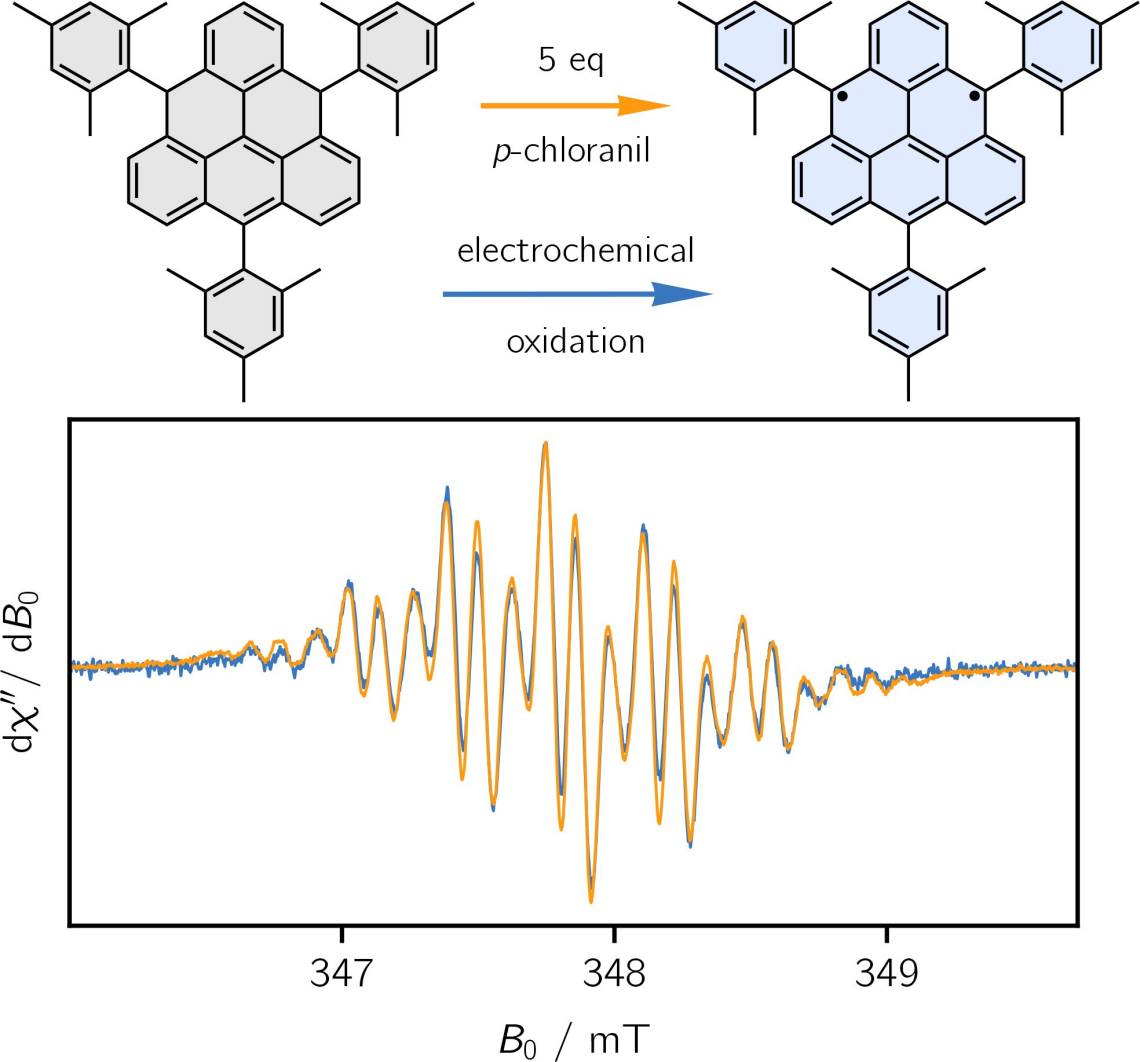
Comparison of the cw‐EPR spectra of the triplet state biradical of mesityl‐substituted triangulene Mes_3_‐Tr obtained by chemical and electrochemical oxidation. The chemical oxidation was carried out by adding 5 equivalents of *p*‐chloranil to a 1 mM solution of the triangulene precursor in toluene, while the electrochemical oxdidation was carried out on a 0.5 mM THF solution with 100 mM TBAHFP.

Figure 4 shows a comparison of the room temperature cw‐EPR spectra of the triangulene triplet state biradical (Mes_3_‐Tr) obtained by chemical and electrochemical oxidation of solutions of the corresponding dihydro‐precursor. The spectra were recorded using a modulation frequency of 100 kHz, modulation amplitudes of 0.01–0.02 mT, and microwave powers <1 mW. In Ref. [11], the chemical oxidation was carried out in toluene using a 0.5–1 mM solution of the precursor with one equivalent of *p*‐chloranil. Under these conditions, the oxidation is slow, requiring at least 6 hours for completion before a triplet spectrum is obtained. Alternatively, the reaction can be sped up by working at higher oxidant concentrations (e. g. 5 equivalents),^[11]^ which requires a waiting time of less than an hour and yields the spectrum shown in Figure 4 (orange line).

The electrochemical oxidation was carried out on a 0.5 mM solution of the dihydro‐precursor in THF with 100 mM TBAHFP. While chemical oxidation is slow in this case, an identical spectrum is obtained by electrochemical oxidation in only a few minutes. The results from chemical and electrochemical oxidation are in excellent agreement, showing that the SEC‐EPR method can become an important tool for the characterisation of paramagnetic functional organic materials.

## Conclusions

In conclusion, we presented the design and potential applications of an easy‐to‐build X‐band SEC‐EPR setup, with the aim to make the in‐situ investigation of radical species more accessible. Many existing SEC‐EPR cells are expensive, difficult to build, or difficult to handle, which may limit the applicability of the technique.

The presented cell is compatible with standard EPR instrumentation and can be built at low cost with commercially available materials. Only access to a glassblower is required. The characterisation showed that inserting the electrodes only has a minimal effect on the Q‐factor of the cavity, enabling the acquisition of high‐quality EPR data in a short time, even on bench‐top EPR instruments.

The comparison of results from chemical and electrochemical oxidation of a graphene fragment further demonstrated the usefulness of the approach and the suitability of the setup to address highly topical research questions.

## Conflict of Interests

The authors declare no competing interests.

1

## Supporting information

As a service to our authors and readers, this journal provides supporting information supplied by the authors. Such materials are peer reviewed and may be re‐organized for online delivery, but are not copy‐edited or typeset. Technical support issues arising from supporting information (other than missing files) should be addressed to the authors.

Supporting Information

## Data Availability

The data that support the findings of this study are available from the corresponding author upon reasonable request.
