# Supplementary material for: Design of an Electrochemical Cell for Continuous Wave EPR Measurements of Radical Ions
Source: Chemistry. 2024 Oct 29;30(69):e202402719. doi: 10.1002/chem.202402719 (PMC11632404; doi:10.1002/chem.202402719)
Supplement: Supplementary file 1 — Supporting Information [file CHEM-30-e202402719-s001.pdf]

Chemistry–A European Journal

Supporting Information

Design of an Electrochemical Cell for Continuous Wave EPR Measurements of Radical Ions

Dominic K. F. Bruyers and Sabine Richert*

Supporting Information for

Design of an Electrochemical Cell for Continuous Wave EPR Measurements of Radical Ions

Dominic K. F. Bruyers, Sabine Richert *

Institute of Physical Chemistry, University of Freiburg, Albertstraße 21, 79104 Freiburg, Germany

* E-mail: sabine.richert@physchem.uni-freiburg.de

Table of Contents

1 Cyclic voltammetry **S1**

1.1 Comparison of the SEC-EPR cell to a standard CV setup S1

1.2 Experimental procedure S2

1.3 Cyclic voltammograms of anthracene, pyrene, and perylene S3

2 EPR characterization **S4**

2.1 EPR setup and parameters S4

2.2 Additional EPR measurements S4

1 Cyclic voltammetry

1.1 Comparison of the SEC-EPR cell to a standard CV setup

To characterise the performance of the SEC-EPR cell for standard electrochemical measurements, cyclic voltammograms were recorded and compared to those measured in a commercial CV-cell under identical conditions. Note that the conditions were optimised for the EPR measurements and are thus far from ideal for CV measurements.

Figure S1 shows a comparison of the data obtained for ferrocene in the SEC-EPR cell and a commercial CV-cell from ALS Japan. The data were recorded using a scan speed of 100 mV/s in THF with 100 mM of tetrabutylammonium hexafluorophospate (TBAHFP) as the electrolyte. In all cases, the potential was referenced against the half-wave potential of the ferrocen/ferrocenium redox couple, which was set to 0 V.

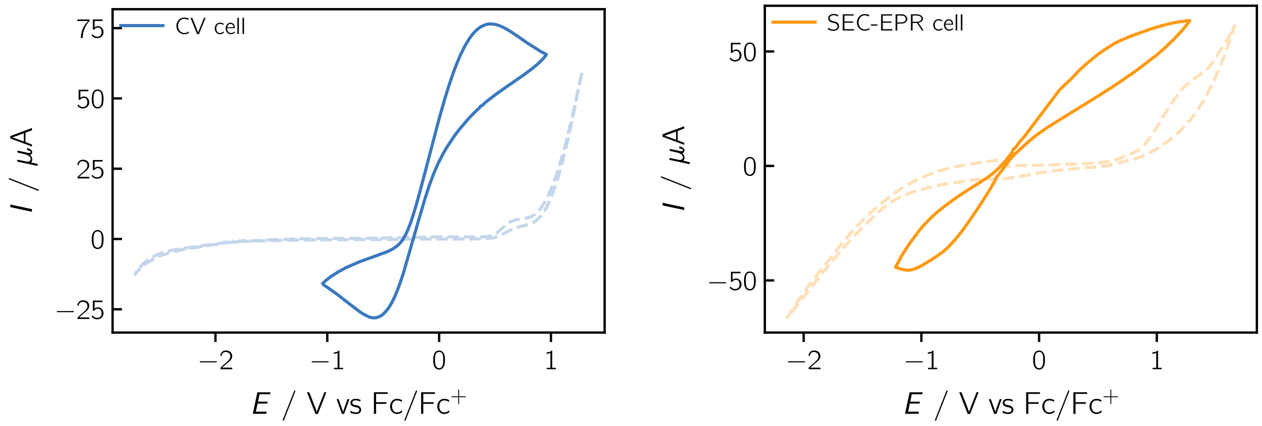

Figure S1: Cyclic voltammogram (100 mV/s) of a 0.2 mM ferrocene solution in THF with 100 mM TBAHFP recorded in a commercial CV-setup (*left*) and in the presented SEC-EPR cell (*right*). The light dotted curve shows the background measured without the analyte.

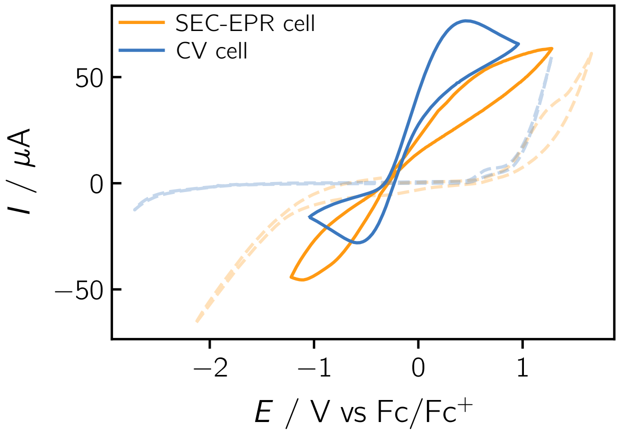

Figure S2: Direct comparison of the two voltammograms.

It can be seen from these figures that the stability window of the electrolyte does not only depend on the solvent (*here:* THF) but also on the working electrode: the platinum electrode used in the SEC-EPR cell provides a window that is about 1-1.5 V narrower than that of the glassy carbonn working electrode used in the commercial setup.[1]

Further, the quality of the CV-data recorded in a standard CV-setup shows, that the chosen conditions are by far not optimal for CV measurements. This is not surprising as the conditions were optimised for

the EPR measurements and, as pointed out in the main part, electrochemical and EPR measurements have vastly different requirements. The electrochemical characterisation of the molecules selected for the measurements in the main part is possible under these conditions, but the results will not be optimal. For instance, we use THF as the solvent to achieve a better Q-value of the EPR cavity and therefore a higher sensitivity in EPR measurements, but THF is by far not the best solvent for CV measurements on the presented molecules. In addition, the commercial CV-setup and the SEC-EPR cell differ in the following aspects:

1. The electrodes in the SEC-EPR cell are simple wires (small surface)
2. The SEC-EPR cell uses a Pt working electrode instead of a glassy carbon electrode
3. The working electrode in the SEC-EPR cell has a very small surface (also compared to the counter and reference electrodes)
4. A different reference electrode is used in the SEC-EPR cell (Ag/AgCl pseudo-reference)

These experimental differences are reflected in the shape of the measured CV-curves: In the CV-cell, the oxidation peak is about three times as high, compared to the reduction peak since diffusion is facilitated (and the solution is moved due to the inert gas being led over it); in the SEC-EPR cell, the oxidation peak is only about 1.5 higher than the corresponding reduction peak since the cell is sealed and the volume around the working electrode is much smaller.

1.2 Experimental procedure

The cyclic voltammograms (CVs) were recorded using a commercial CV-setup for small volumes (VC-4) from ALS Japan. The CV-cell was equipped with a glassy carbon working electrode and a platinum counter electrode. A non-aqueous (100 mM TBAHFP in THF) silver electrode (Ag/Ag^{+}, RE-7 from ALS Japan) was used as the reference.

The CVs of solutions of the analytes in THF using 100 mM TBAHFP as the electrolyte were recorded with a scan speed of 100 mVs^{-1}, unless stated otherwise. For the measurements, a spatula tip of the analyte was added to the electrolyte solution that had been measured before and the CV was recorded with the same settings. To receive a symmetric shape of the analyte voltammogram, the potential window was reduced in width. After the measurement, a spatula tip of ferrocene was added to the solution and the CV was recorded again. The data were then referenced against the ferrocenium/ferrocene redox couple by setting the half-wave potential of Fc/Fc^{+} to 0 V.

The procedure can be seen in Figure S3. After the measurement of anthracene (blue graph), ferrocene was added to the solution (yellow graph) to reference the potential. The measurements in the SEC-EPR cell were performed analogously. The setup is described in detail in the main part.

The concentrations of the analytes in the solutions were determined from their respective UV-vis spectra recorded after the measurement. To calculate the concentrations, the following molar absorption coefficients were used: for anthracene $\varepsilon_{323\ \mathrm{nm}} = 3400\ \mathrm{cm^{-1}M^{-1}}$,[2] for pyrene $\varepsilon_{335\ \mathrm{nm}} = 55000\ \mathrm{cm^{-1}M^{-1}}$,[2] for perylene $\varepsilon_{408\ \mathrm{nm}} = 28000\ \mathrm{cm^{-1}M^{-1}}$,[2] and for triangulene $\varepsilon_{410\ \mathrm{nm}} = 20000\ \mathrm{cm^{-1}M^{-1}}$.[3] Since the solutions were a mixture of the analyte and ferrocene, the UV-vis spectra had to be corrected. The concentration of ferrocene was determined with a molar absorption coefficient of $\varepsilon_{520\ \mathrm{nm}} = 11\ \mathrm{cm^{-1}M^{-1}}$.[4]

1.3 Cyclic voltammograms of anthracene, pyrene, and perylene

The following figures show the cyclic voltammograms of anthracene, pyrene and perylene recorded both in a standard CV-cell and the presented SEC-EPR cell. As mentioned above, the conditions are far from ideal for CV-measurements in general, but the comparison shows that the performance of the SEC-EPR cell is not much worse compared to a standard cell. Note that a direct comparison is invalid due to the design differences pointed out above.

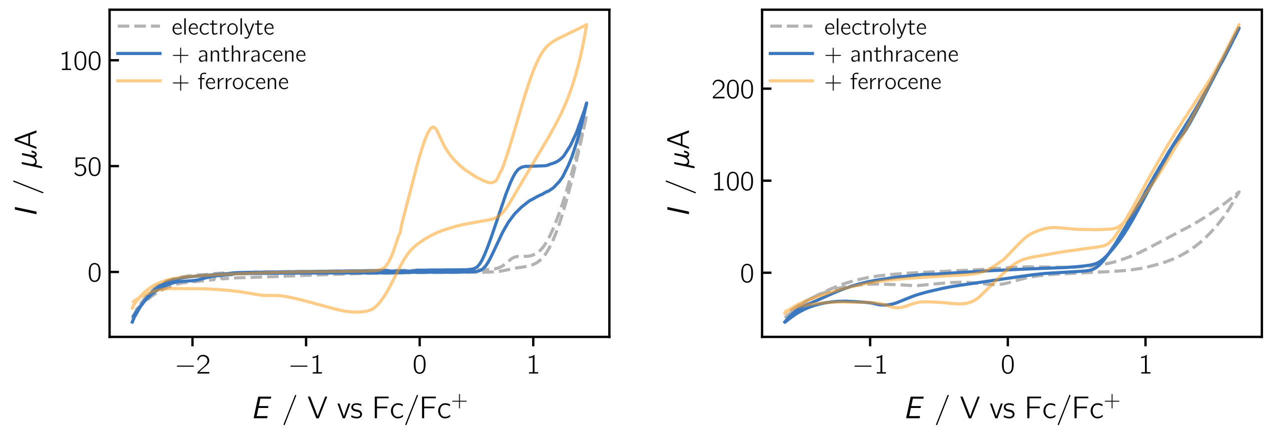

Figure S3: Cyclic voltammogram (100 mV/s) of a 10 mM anthracene solution in THF with 100 mM TBAHFP recorded in a commercial CV-setup (*left*) and in the presented SEC-EPR cell (*right*).

The cyclic voltammogram of anthracene in the commercial setup is consistent with literature data.[5] In the SEC-EPR setup the shape of the CV looks different due to the reasons stated above. Still, the increase in current starts at the same potential as in the commercial setup.

The CV of pyrene also yielded the expected result. As stated in literature, the successful measurement of pyrene is only possible in special solvents like EC:DMC (3:7 volume ratio) which provide a enlarged stability window.[6] In this work, optimised parameters for EPR were used that made the measurement of a classic CV shape impossible.

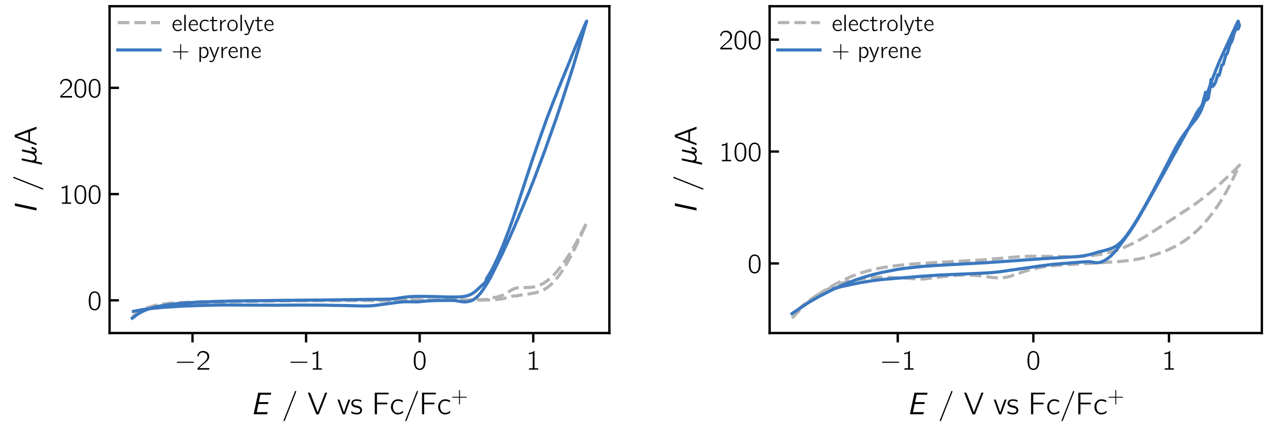

Figure S4: Cyclic voltammogram (100 mV/s) of a 1 mM pyrene solution in THF with 100 mM TBAHFP recorded in a commercial CV-setup (*left*) and in the presented SEC-EPR cell (*right*).

The measurement of perylene yielded a CV that is well known in literature.[6] In the SEC-EPR cell the uneven baseline causes a deviation from the ideal shape of the CV. This could already be observed in the CV of ferrocene in Figure S2.

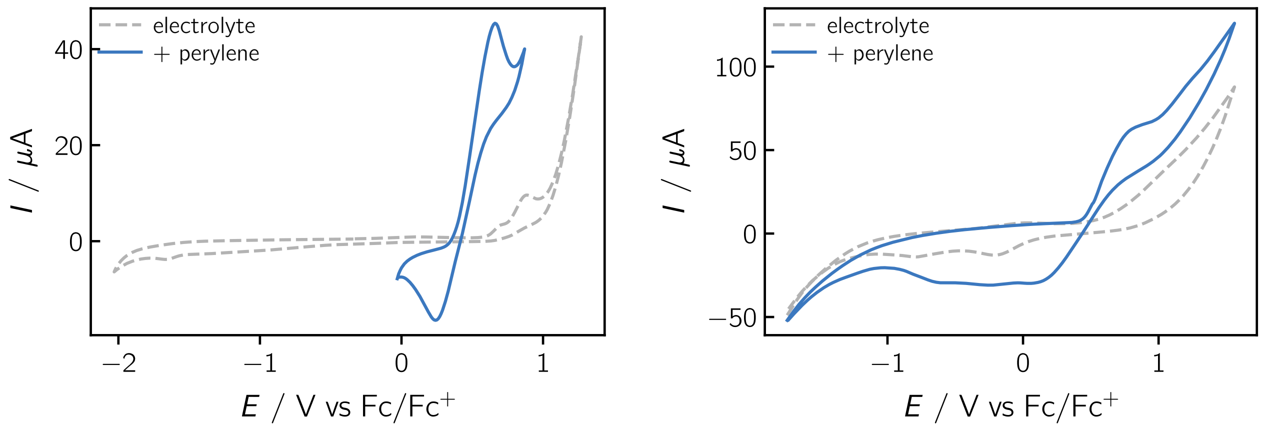

Figure S5: Cyclic voltammogram (100 mV/s) of a 1 mM perylene solution in THF with 100 mM TBAHFP recorded in a commercial CV-setup (*left*) and in the presented SEC-EPR cell (*right*).

2 EPR characterisation

2.1 EPR setup and parameters

To record the EPR spectra presented in the main part, the potential was set to the following values: for anthracene 1.6 V, for pyrene 2.2 V, for perylene 0.7 V, for triangulene −1.8 V. These potentials were measured against the Ag/AgCl pseudo reference electrode and are not corrected against the ferrocen/ferrocenium redox pair. This correction would imply a potential shift of about −0.5 V to −0.7 V.

2.2 Additional EPR measurements

To compare the quality of the EPR data recorded in a standard EPR cell and the presented SEC-EPR cell, additional cw EPR measurements were performed with a stable radical (TEMPO).

The EPR spectra were recorded at the X-band (9.75 GHz) at room temperature on a Bruker EMXnano benchtop EPR spectrometer using the quartz EPR tube of the presented SEC-EPR cell (inner diameter of 1.2 mm; detailed specifications can be found in the main part). The modulation frequency was set to 100 kHz and the modulation amplitude to 0.1 mT at a microwave power of 1 mW (20 dB). The recorded, background-corrected, spectra were frequency-corrected to 9.75 GHz and field-corrected using a carbon fibre standard with $g = 2.002644$.[7]

Different concentrations of TEMPO between 50 and 200 μM were used. Without the electrodes, a cavity Q-factor of 5400 is obtained, whereas, with the electrodes, the Q-factor amounts to 4900. Consequently, no major difference in the signal intensity is expected, as shown in Figure S6.

Figure S7 shows EPR spectra of TEMPO in THF measured at different dilutions in the SEC-EPR-cell down to a concentration of 50 μM.

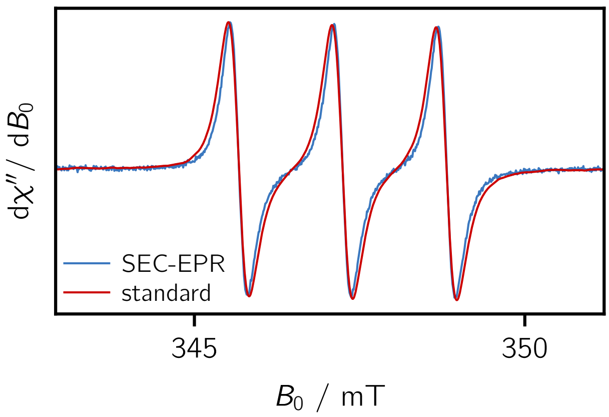

Figure S6: EPR spectra of a 200 μM solution of TEMPO in THF. The spectrum recorded in the assembled SEC-EPR cell (blue line) is compared to the spectrum obtained when performing the measurement in a comparable standard quartz tube (inner diameter 1.2 mm).

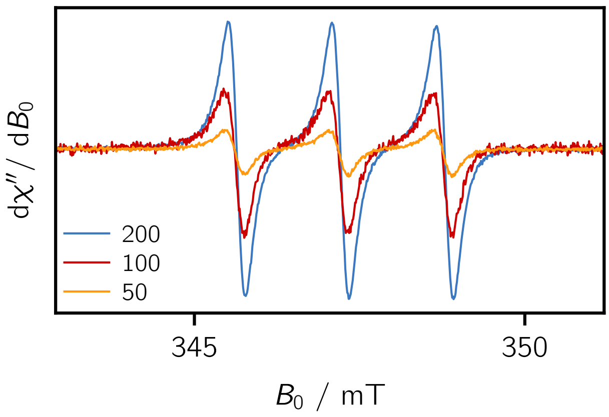

Figure S7: EPR spectra of a solution of TEMPO in THF at different concentrations (200 μM, 100 μM and 50 μM) recorded in the assembled SEC-EPR cell.

References

[1] Coustan, L.; Shul, G.; Bélanger, D. Electrochemical behavior of platinum, gold and glassy carbon electrodes in water-in-salt electrolyte. *Electrochem. Commun.* **2017**, *77*, 89–92.

[2] Berlman, I. *Handbook of Florescence Spectra of Aromatic Molecules*; Elsevier, 2012.

[3] Valenta, L.; Mayländer, M.; Kappeler, P.; Blacque, O.; Šolomek, T.; Richert, S.; Juríček, M. Trimesityltriangulene: a persistent derivative of Clar's hydrocarbon. *Chem. Commun.* **2022**, *58*, 3019–3022.

[4] Bozak, R. *Photochemistry in the Metallocenes*; Wiley Online Library, 1971; Vol. 8; pp 227–244.

[5] Xue, T.; Zhao, D.; Hao, T.; Li, X.; Wang, T.; Nie, J. Synthesis, one/two-photon optical and electrochemical properties and the photopolymerization-sensitizing effect of anthracene-based dyes: Influence of the donor groups. *New J. Chem.* **2019**, *43*, 6737–6745.

[6] Bachman, J.; Kavian, R.; Graham, D.; Kim, D.; Noda, S.; Nocera, D.; Shao-Horn, Y. Electrochemical polymerization of pyrene derivatives on functionalized carbon nanotubes for pseudocapacitive electrodes. *Nat. Commun.* **2015**, *6*, 7040.

[7] Herb, K.; Tschaggelar, R.; Denninger, G.; Jeschke, G. Double resonance calibration of *g* factor standards: carbon fibers as a high precision standard. *J. Magn. Reson.* **2018**, *289*, 100–106.
